# Effects of calcium Ionophore A23187 on the apoptosis of hepatic stellate cells stimulated by transforming growth factor-β_1_

**DOI:** 10.1186/s11658-017-0063-z

**Published:** 2018-01-02

**Authors:** Yanan Li, Yu Yan, Fang Liu, Ming Wang, Fumin Feng, Yonghong Xiao

**Affiliations:** 1 Department of School of Public Health, North China University of Science and Technology, Hebei, Tang Shan, 063000 China; 2 Department of School of Basic Medical Science, North China University of Science and Technology, Hebei, Tang Shan, 063000 China

**Keywords:** Hepatic fibrosis, Hepatic stellate cells, Calcium ionophore A23187, Apoptosis

## Abstract

**Background:**

Our previous study showed that during in vitro experiments changes in calcium concentration were associated with apoptosis. We presumed that the calcium ion might play a role as intermediate messenger for apoptosis-related genes. No such evidence has been reported in the literature. Here, we investigate the effect of calcium ionophore A23187 on the apoptosis of rat hepatic stellate cells (HSCs) stimulated by transforming growth factor-β_1_ (TGF-β_1_) to explore the mechanism of apoptosis through the endoplasmic reticulum stress pathway.

**Methods:**

The apoptotic rate was determined using flow cytometry. The changes in Ca^2+^ level in HSCs were examined with laser confocal microscopy. The expressions of caspase-12 GRP78 and caspase-9 were assayed via western blot.

**Results:**

The respective apoptosis rates for the blank group, the TGF-β_1_ group and the TGF-β_1_ + low, medium and high dose calcium ionophore A23187 groups were 3.40 ± 0.10%, 1.76 ± 0.12%, 5.86 ± 0.31%, 11.20 ± 0.48% and 15.08 ± 0.75%, with significant differences between the groups (*p* < 0.05). The concentration of Ca^2+^and the expression of the GRP78, caspase-9 and caspase-12 proteins significantly increased with increasing calcium ionophore A23187 doses (*p* < 0.05).

**Conclusion:**

Calcium ionophore A23187 increased intracellular Ca^2+^ and activated endoplasmic reticulum stress, which promoted HSC apoptosis.

## Introduction

Hepatic fibrosis (HF) is a reversible wound-healing response to all etiologies of liver disease, including chronic viral hepatitis, alcohol consumption, fatty liver disease, cholestasis and autoimmune hepatitis [1]. Hepatic stellate cells (HSCs) are central to the development of HF [2].

When HSCs are activated, they transform into myofibroblasts and secrete a large volume of extracellular matrix (ECM). Transforming growth factor beta-1 (TGF-β_1_) can activate HSCs and increase the expression of various fibrosis factors that playan important role in HF [3].

Promoting HSC apoptosis can reduce or even reverse HF, and induction of the activation of HSC apoptosis as a therapeutic strategy is a research hotspot [4]. Endoplasmic reticulum stress (ERS) is a recently discovered pathway of apoptosis. It includes the non-folding protein reaction, Ca^2+^ initiation signals and other ERS-specific mechanisms. It can activate caspase-12 and mediate caspase-12 cleavage of caspase-9 and other downstream effects of protease, eventually leading to apoptosis [5].

The endoplasmic reticulum (ER) is the main site of the intracellular Ca^2+^ reserve and Ca^2+^ signal transduction [6]. Calcium ionophore A23187 is a calcium mobilizer that plays a critical role in cell apoptosis [7]. Therefore, this study to investigate the effects of calcium ionophore A23187 on the apoptosis of HSC stimulated by transforming growth factor-β_1_ to provide a theoretical basis for the treatment of HF.

## Materials and methods

### Cell Strains,Cell culture and cell grouping and treatment

The hepatic stellate cell line CFSC isolated from carbon tetrachloride-stimulated rats was provided by Professor Greenwell from the cell bank of George Washington University in the United States [8].The cells stored in liquid nitrogen were cultured in Dulbecco’s modified Eagle’s medium (DMEM; BI) supplemented with 6% fetal bovine serum (FBS; BI), 100 U/ml penicillin and 100 μg/ml streptomycin in humidified air at 37 °C with 5% CO_2_ (Thermo). When the cells reached 80% confluence, they were dislodged by trypsinization and seeded in culture bottles.The cells were divided into the following groups: blank, TGF-β_1_ (5 ng/ml), TGF-β_1_ (5 ng/ml) + low,TGF-β_1_ (5 ng/ml) + middle and TGF-β_1_ (5 ng/ml) + high, with low, middle and high referring to the dose of calcium ionophore A23187. The blank group was cultured in DMEM without FBS for 48 h. The TGF-β_1_ group was cultured with 5 ng/ml TGF-β_1_ (Glico) for 48 h. The TGF-β_1_ + low, middle, and high dose calcium ionophore A23187 groups were cultured with 5 ng/ml TGF-β_1_ to which 1, 2 and 4 μM A23187 (Cayman) were added, respectively. The changes to each index were measured after 24 h.

### Annexin V/PI analysis of HSC apoptosis rate

After treatment with A23187, the cells were washed with cold PBS three times, dislodged by trypsinization, centrifuged, and then washed with cold PBS three times. After setting aside the blank group of cells as a negative control, the other groups were resuspended in 195 μl of annexin V binding buffer, incubated with 5 μl of FITC-conjugated annexin V (BD), and mixed gently for 30 min in the dark. After centrifugation, the supernatant was discarded, the cells were resuspended in 195 μl of annexin V binding buffer, incubated with 5 μL of propidium iodide (BD), and mixed gently. Finally, the cells were examined using flow cytometry (FACSCalibur).

### Cellular Ca^2+^ imaging and laser scanning confocal microscopy

The cells were conventionally cultured, dislodged by trypsinization, and seeded into a small dish for confocal microscopy. HSCs were harvested after the appropriate treatment. The culture medium was discarded, the cells were washed with cold PBS three times and incubated with 400 μl Fluo-3/AM (KPL) for 40 min at 37 °C in the dark. Then the cells were washed with cold PBS three times, ~1 ml culture medium was added, and the dish was incubated for 20 min. Changes in cellular Ca^2+^ were monitored using a laser scanning confocal microscope (FV10i).

### Western blot assay for GRP78, C-Caspase-12 and C-Caspase-9 protein expression

The cells were treated with calcium ionophore A23187 for 24 h, after which the original culture medium was discarded, and the cells were washed with cold PBS twice or three times. The culture dish was set on ice and ~200 μl cell lysis buffer was added (Protease inhibitor: RIPA = 1:250). The cells were adequately cleaved for 10–30 min and then centrifuged (12,000 r/min; 4 °C) for 15 min.

A BCA Protein Assay kit (MultiSciences) was used to determine the protein concentration. The cells were boiled for 5 min at 100 °C and stored at −20 °C. A 10% SDS-PAGE gel preparation kit (Beyotime Biotechnology) was used to assay GRP78 and caspase-9 protein expression. After blocking, the turn wet method was used to transfer the cells to PVDF membrane and then GRP78 (1:1000; Arigo), C-caspase-12 (1:1000; Arigo) and C-caspase-9 (1:1000; Wuhan) antibodies were added at 4 °C overnight. The membrane was washed with TBST three times, the secondary antibody was added, and the membrane was incubated at room temperature for about 1 h and washed with TBST three times. Development used equivalently-mixed ECL luminescence reagents A and B. Images were taken using an E-Gel Imager (Universal Hood II).

### Statistical analysis

Results were described as means ± SD of at least three independent experiments. Comparisons across time points were performed with one-way analysis of variance to detect the main effect differences using 17.0 SPSS statistical analysis software. Comparison between two means was performed with LSD-t to detect the main effect differences. In all cases, *p* < 0.05 was considered to be statistically significant.

## Results

### Flow cytometry to determine the apoptosis rate in the treated cells

**Fig. 1:**
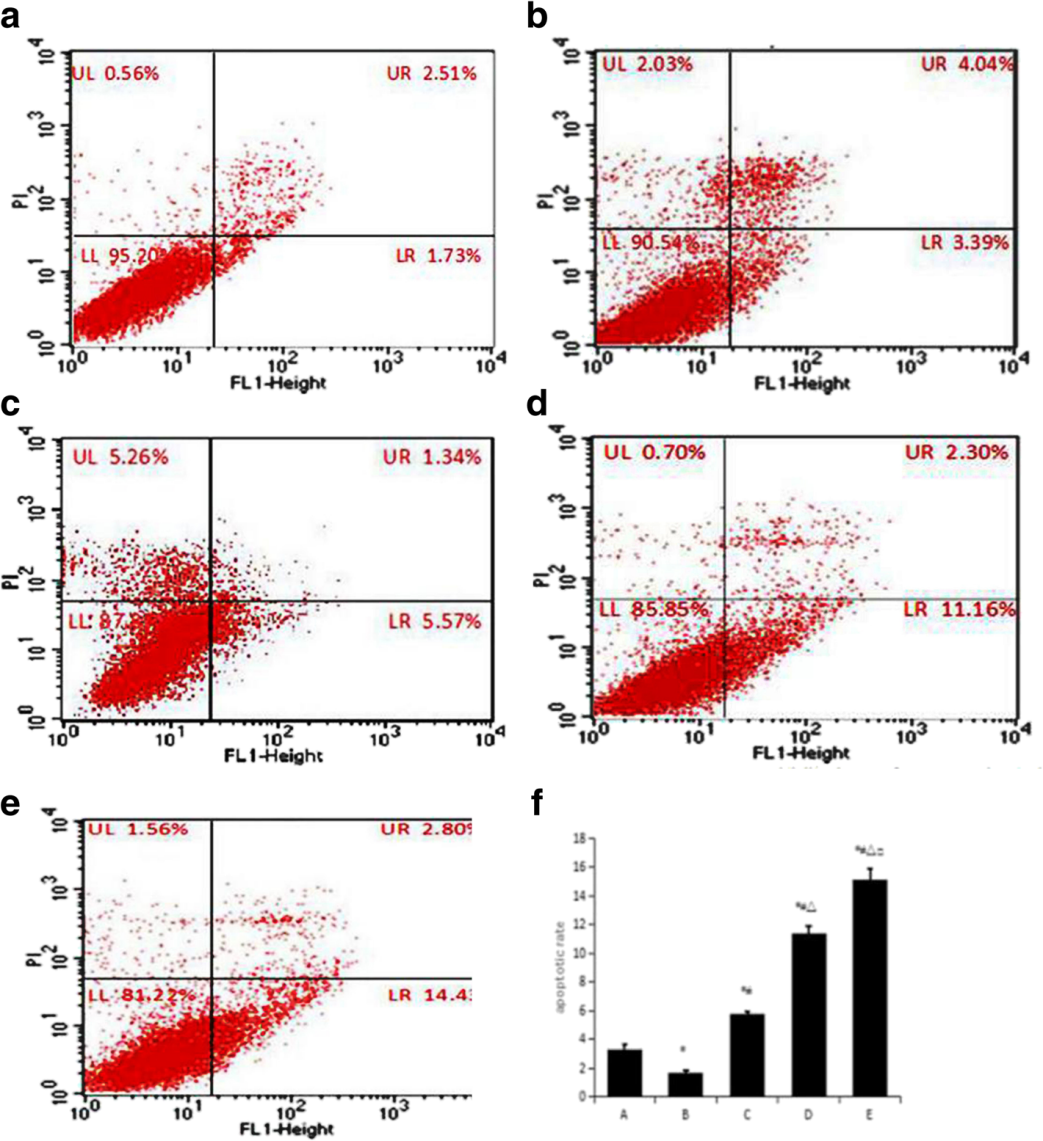
Flow cytometry scatter plots and histogram plot of different treatments leading to apoptosis of rat hepatic stellate cells. **a** – blank group; **b** – TGF-β_1_ group; **c** – TGF-β_1_ + low dose of calcium ionophore A23187 group; **d** – TGF-β_1_ + middle dose of calcium ionophore A23187 group; **e** – TGF-β_1_ + high dose of calcium ionophore A23187 group. **f** – Changes in HSC apoptosis rate in the different treatment groups ($\overline{x}$±s) expressed as a percentage. Denoted A through E as above. *p < 0.05 compared to the blank group; ^#^*p* < 0.05 compared to the TGF-β_1_ group; ^△^p < 0.05 compared to the TGF-β_1_ + low dose of calcium ionophore A23187 group; ^□^p < 0.05 compared to the TGF-β_1_ + middle dose of calcium ionophore A23187 group

Flow cytometry was used to determine the apoptosis rate for HSCs (Fig. 1). Comparisons were performed with one-way analysis of variance to detect group differences. The apoptosis rate of the TGF-β_1_ group was 1.76 ± 0.12%, which was lower than that of the blank group (3.40 ± 0.10%). The difference was statistically significant (*p* < 0.05). The apoptosis rates of the TGF-β_1_ + low, middle and high dose calcium ionophore A23187 groups were respectively 5.86 ± 0.31%, 11.20 ± 0.48% and 15.08 ± 0.75%, with significant differences between the groups (*p* < 0.05; Fig. 1f).

### Effects of calcium Ionophore A23187 on Ca^2+^ levels in rat hepatic stellate cells following stimulation with transforming growth factor-β1

**Fig. 2:**
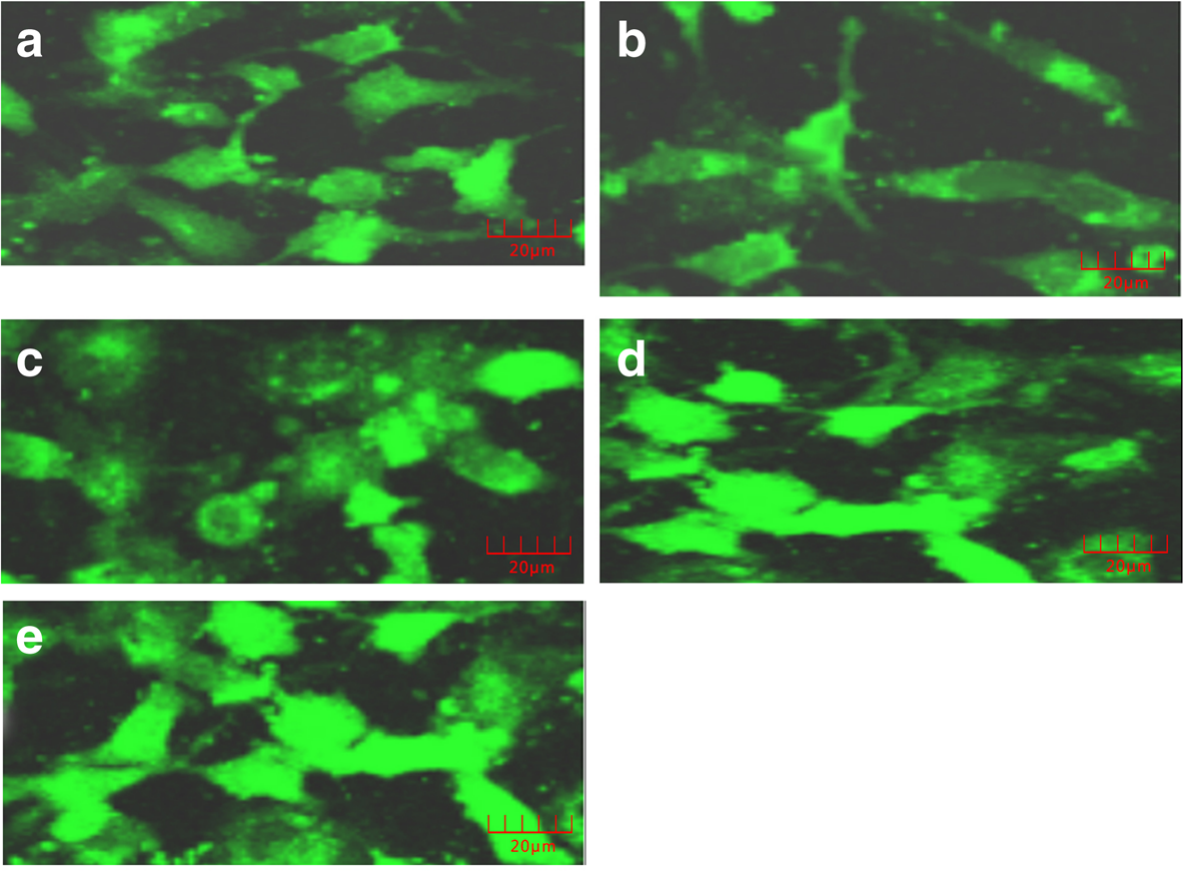
Laser confocal microscopy images of the changes in Ca^2+^ fluorescence intensity. **a** – blank group; **b** – TGF-β_1_ group; **c, d** and **e** – TGF-β_1_ + low, middle and high dose of calcium ionophore A23187 groups

**Fig. 3:**
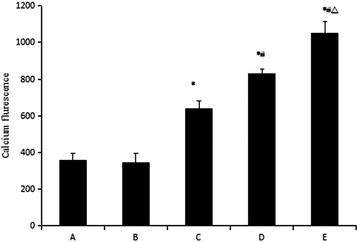
Effect of different treatments on HSC calcium fluorescence. A – blank group; B – TGF-β_1_ group; C, D and E – TGF-β_1_ + low, middle and high dose of calcium ionophore A23187 groups. *p < 0.05 compared to the blank group; ^#^*p* < 0.05 compared to the TGF-β_1_ group; ^△^*p* < 0.05 compared to the TGF-β_1_ + low dose of calcium ionophore A23187 group

Laser confocal microscopy was used to determine the calcium fluorescence in HSCs (Fig. 2). The blank group and TGF-β_1_ group presented weak fluorescence in the cytoplasm (Fig. 2a and b; *p* > 0.05). Adding the calcium ionophore A23187 after TGF-β_1_ stimulation led to strong fluorescence (*p* < 0.05; Fig. 2c–e). The LSD method showed that the intracellular Ca^2+^ concentration of the two pairs was statistically significant (*p* < 0.05; Fig. 3).

### The expression of GRP78, C-caspsase-12 and C-caspase-9 in different treatment groups

**Fig. 4:**
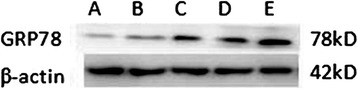
GRP78 protein expressing in different HSC groups as measured using western blot. A – blank group; B – TGF-β_1_ group; C, D and E – TGF-β_1_ + low, middle and high dose of calcium ionophore A23187 groups

Protein gel electrophoresis of GRP78 expression in the different treatment groups of HSC (Fig. 4). The mean grey values of GRP78 protein the blank group and the TGF-β_1_ group were 0.34 ± 0.05 and 0.31 ± 0.03, respectively, and the difference was not statistically significant (*p* > 0.05). The mean grey values of the low, middle and high dose calcium ionophore A23187 groups were 0.46 ± 0.06, 0.62 ± 0.03 and 0.79 ± 0.01, respectively.

**Fig. 5:**
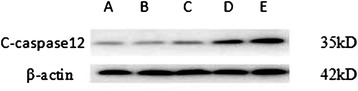
C-caspase-12 protein expression in the different HSC groups as measured using western blot. A – blank group; B – TGF-β_1_ group; C, D and E – TGF-β_1_ + low, middle and and high dose of calcium ionophore A23187 groups

As the dose of A23187 increased, the expression of GRP78 protein also increased in the groups, and the differences between the two were statistically significant (*p* < 0.05; Fig. 7). The protein gel electrophoresis of C-caspase-12 in different treatment groups is shown in Fig. 5. When compared to the blank group, C-caspase-12 change in the TGF-β_1_ group was not obvious (*p* > 0.05). The mean grey values of the low, middle and high dose calcium ionophore A23187 groups were 0.48 ± 0.03, 0.66 ± 0.06 and 0.90 ± 0.03, respectively. As the dose of A23187 increased, the expression of C-caspase-12 protein also increased in the groups, and the differences between the two were statistically significant (*p* < 0.05).

**Fig. 6:**
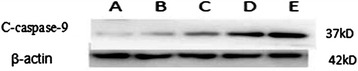
C-caspase-9 protein in the different HSC groups as measured using western blot. A – blank group; B – TGF-β_1_ group; C, D and E – TGF-β_1_ + low, middle and high dose calcium ionophore A23187 groups

**Fig. 7:**
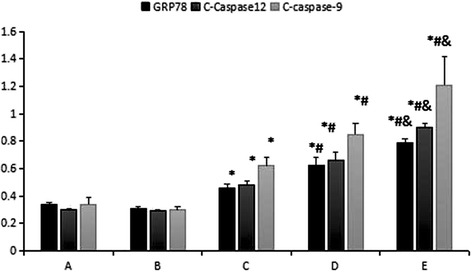
Expressions of GRP78, C-caspase-12 and C-caspase-9 protein in the different treatment groups of hepatic stellate cells. A – blank group; B – TGF-β_1_ group; C, D and E – TGF-β_1_ + low, middle and high dose calcium ionophore A23187 groups. *p < 0.05 compared to the blank group; ^#^p < 0.05 compared to the TGF-β_1_ group; ^&^p < 0.05 as compared to the TGF-β_1_ + low dose of calcium ionophore A23187 group

Protein gel electrophoresis of C-caspase-9 expression in the different treatment groups of HSCs is shown in Fig. 6. The mean grey values of C-caspase-9 protein for the blank group and the TGF-β_1_ group were 0.34 ± 0.05 and 0.30 ± 0.02, respectively. The difference was not statistically significant (*p* > 0.05). The mean grey values of the low, middle and high dose calcium ionophore A23187 groups were 0.62 ± 0.06, 0.85 ± 0.08 and 1.21 ± 0.21, respectively. As the dose of A23187 increased, the expression of C-caspase-9 protein also increased in the groups, and the difference between the two was statistically significant (*p* < 0.05; Fig. 7).

## Discussion

Ca^2+^ is an important secondary messenger in cells. It is involved in the regulation of various physiological functions, such as cell growth, proliferation, differentiation and apoptosis [9]. In the resting state, the intracellular Ca^2+^ concentration is maintained at a very low level, and is about 1/10,000 of the extracellular Ca^2+^ concentration. In response to a suitable stimulus, the intracellular Ca^2+^ concentration increases.

The calcium ionophore A23187 is the chain ion carrier. External lipophilic complexes of Ca^2+^ A23187 form at a 1:2 ratio, with two molecules of A23187 end-to-end. The transport of Ca^2+^ is performed by the carrier complex in the membrane. The effect is different in different cells, which indicates that the signal to the channel is also different. For instance, increased Ca^2+^ as a secondary messenger can promote protein kinase C and other calcium-dependent protein kinase activation, and catalyze intracellular phosphorylation of various proteins to initiate cell activation and proliferation. A23187 can rapidly increase intracellular Ca^2+^ concentration in HL-60 cells, disrupting the intracellular Ca^2+^ steady-state balance to initiate cell apoptosis [10].

To investigate the effect of A23187 on apoptosis in rat HSCs, the cells were stimulated with TGF-β_1_. Electron microscopy showed that the cytoplasm had numerous collagen fibers and the nucleus was in the split phase, suggesting that myofibroblasts participated in the development of HF, mainly through their own abnormal proliferation. Furthermore, numerous collagen fibers contain collagen secretion from the extracellular matrix (ECM).

Activated HSCs are the primary source of ECM in HF. They play a key role in its formation and development. TGF-β_1_ is the most powerful fibrosis promoting factor that can be used to stimulate HSCs to create a model of HF in vitro. TGF-β_1_ can be used for mechanism research into the regulation of HSC gene expression and the potential of related pathways as therapeutic modalities [3].

The cells were stimulated with TGF-β_1_ and then different doses (1, 2 and 4 μM) of A23187 were added. The effect of A23187 on the apoptotic rate was detected using flow cytometry. The apoptosis rate of HSCs was increased with an increase in calcium ionophore concentration. A23187 can form stable complexes with Ca^2+^ and open the calcium channel on the cell membrane, leading to increase in intracellular calcium concentration. In this study, Fluo-3/AM-loaded cells exposed to low, middle and high doses of A23187 were used to observe the intracellular Ca^2+^ steady-state imbalance. Ca^2+^ elevation could activate the ERS pathway, which could be the cause of apoptosis.

Our study shows that in these HSCs, the intracellular Ca^2+^ homeostasis was mainly maintained through the endoplasmic reticulum (ER). Various factors can cause changes in Ca^2+^ channels on the ER, resulting in Ca^2+^ deprivation or Ca^2+^ overload and activation of the ERS pathway.

GRP78 is an ERS chaperone that plays an important role in maintaining ER protein synthesis, proper protein folding and intracellular calcium homeostasis. It is also an important marker of ERS [11, 12]. GRP78 plays a key regulatory role in ERS and is an important defense mechanism for cells. In this study, the expression of GRP78 protein was detected via immunoblotting. Different doses of A23187 led to high expression of GRP78 protein, suggesting that intracellular ERS was activated.

Ca^2+^ initiates an early step in apoptosis, which is closely linked to the ER. ERS has a unique caspase-12 pathway. Caspase-12 belongs to the caspase family and is considered to be a specific apoptotic signal in the ERS apoptotic pathway. It is activated by ERS and can mediate mitochondrial-independent apoptosis [13]. Under normal conditions, caspase-12 is located on the ER in the form of inactive zymogens. When ERS occurs, IRE1α activates TRAF2 to activate caspase-12 and caspase-9, causing caspase-mediated apoptosis [14].

Xie et al. [15] induced ER stress in cells of the human hepatocarcinoma cell line Huh-7 with toxic carotene (TG). Western blotting was used to detect procaspase-12, which was found to be significantly associated with TG and apoptosis. Bitko et al. [16] found that apoptosis of A549 human lung epithelial cells caused by respiratory syncytial virus was associated with caspase-12 activation and ERS. Here, we found that low, middle and high doses of A23187 significantly upregulated the expression of intracellular caspase-12 and caspase-9.

## Conclusion

These results suggest that A23187 increases intracellular Ca^2+^, causing intracellular calcium disorders, and activates the ERS pathway to promote HSC apoptosis.

## Abbreviations

DMEM: Dulbecco’s modified Eagle’s medium
ECM: Extracellular matrix
ERS: Endoplasmic reticulum stress
HF: Hepatic fibrosis
HSC: Hepatic stellate cell
TGF-β_1_: Transforming growth factor- β_1_

## References

[1] Pereira TN, Walsh MJ, Lewindon PJ (2010). Paediatric cholestatic liver disease:Diagnosis,assessment of disease progression and mechanisms of fibrogenesis. World J Gastrointest Pathophysio.

[2] Erkan M, Weis N, Pan Z (2010). Organ-,inflammation- and cancer specific transcriptional fingerprints of pancreatic and hepatic stellate cells. Mol Cancer.

[3] Zhou W-c, Zhang Q-B, Liang Q (2014). Pathogenesis of liver cirrhosis. World J Gastroenterol.

[4] Weiskirchen R, Tacke F (2014). Cellular and molecular functions of hepatic stellate cells in inflammatory responses and liver immunology. Hepatobiliary Surg Nutr.

[5] Sharma V, Kaur R, Bhatnagar A (2015). Low-pH-induced apoptosis: role of endoplasmic reticulum stress-induced calcium permeability and mitochondria-dependent signaling. Cell Stress Chaperones.

[6] Montague K, Malik B, Gray AL (2014). Endoplasmic reticulum stress in spinal and bulbar muscular atrophy: a potential target for therapy. Brain.

[7] Liu Y, Han X-j, Liu M-h (2014). Three-day-old human unfertilized oocytes after in vitro fertilization/intracytoplasmic sperm injection can be activated by calcium Ionophore A23187 or strontium chloride and develop to blastocysts. Cell Reprogram.

[8] Yonghong X, Dianwu L. Effect of transforming growth factor - β1 antibody on intracellular calcium concentration in hepatic stellate cells. J FourthMilMed Univ. 2005;26(17):1558-561.

[9] Bahar E, Kim H, Yoon H (2016). ER stress-mediated signaling: action potential and Ca2+ as key players. Int J Mol Sci.

[10] Yang K, Qiang L, Yaining Z (2007). A23187 induced HL-60 differentiation into dendritic cells. J Sichuan University (Medical Sciences).

[11] Axel H. Schönthal.Endoplasmic reticulum stress: its role in disease and novel prospects for therapy. Scientifica (Cairo). 2012:857516. 10.6064/2012/857516 PMC3820435 24278747

[12] Cho H, Wu M, Zhang L (2013). Signaling dynamics of palmitate-induced ER stress responses mediated by ATF4 in HepG2 cells. BMC Syst Biol.

[13] Sun Y, Liu G, Song T (2008). Upregulation of GRP78 and caspase-12 in diastolic failing hea. Acta Biochim Pol.

[14] Kim EM, Shin EJ, Choi JH (2010). Matrix metalloproteinase-3 is increased and participates in neuronal apoptotic signalingdownstream of caspase-12 during endoplasmic reticulum stress. J Biol Chem.

[15] Xie Q, Khaoustov VI, Chung CC (2002). Effect of tauroursodeoxycholic acid on ER stress_induced Caspase-12 activation. Hepatology.

[16] Nakagawa T, Zhu H, Morishima N (2000). Caspase-12 mediates ER_specific apoptosis and cytotoxicity by amyloid_β. Nature.

